# Studying the effect of alpha-synuclein and Parkinson’s disease linked mutants on inter pathway connectivities

**DOI:** 10.1038/s41598-021-95889-5

**Published:** 2021-08-11

**Authors:** Sagnik Sen, Ashmita Dey, Ujjwal Maulik

**Affiliations:** Computer Science and Engineering, Jadavpur University, Kolkata, India

**Keywords:** Computational biology and bioinformatics, Protein analysis

## Abstract

Parkinson’s disease is a common neurodegenerative disease. The differential expression of alpha-synuclein within Lewy Bodies leads to this disease. Some missense mutations of alpha-synuclein may resultant in functional aberrations. In this study, our objective is to verify the functional adaptation due to early and late-onset mutation which can trigger or control the rate of alpha-synuclein aggregation. In this regard, we have proposed a computational model to study the difference and similarities among the Wild type alpha-synuclein and mutants i.e., A30P, A53T, G51D, E46K, and H50Q. Evolutionary sequence space analysis is also performed in this experiment. Subsequently, a comparative study has been performed between structural information and sequence space outcomes. The study shows the structural variability among the selected subtypes. This information assists inter pathway modeling due to mutational aberrations. Based on the structural variability, we have identified the protein–protein interaction partners for each protein that helps to increase the robustness of the inter-pathway connectivity. Finally, few pathways have been identified from 12 semantic networks based on their association with mitochondrial dysfunction and dopaminergic pathways.

## Introduction

One of the common neurodegenerative disorders (NDs) is Parkinson’s disease (PD) which has been reported to be associated with the protein alpha-synuclein(Asyn)^1,2^. Asyn$_s$ are usually known for their robust expression within lewy bodies (LB). For example, the association between expression levels of the Asyn especially through the Lewy Bodies, and functional disorder of the dopaminergic neuron are reported^3^. Propensity of the Asyn towards aggregation might be responsible for functional disorder of its protein partners^4^. These genetic aberrations due to mutations can lead to functional modifications. For example, the EGFR1 signaling pathway is highly dependent on the activities of the asyn proteins. Any mutational modification can lead to functional changes in EGFR1 signaling pathways. In 1997, a point mutation at G209A has been reported. The substitution has resulted in point mutation A53T which is an early onset mutation linked with PD. However, this mutation is highly debatable as threonine can naturally be identified at rodent synuclein homologs, followed by two more mutations Viz., E46K, and A30P. The observation has justified the occurrence of functional similarity and pathway orchestrations associated with synuclein homologs and paralogs. Early-onset and late-onset mutations Viz., A53T, E46K, H50Q and G51D are reported in^5^. Usually, alpha-synuclein has three domains. Among them, A53T, E46K and H50Q are shown to enhance the aggregation propensity^6,7^ whereas G51D is associated with slower rate of aggregation. Similarly , non-beta component of Alzheimer’s disease amyloid plaque (NAC) domain i.e., residue range 61–95 plays significant role during aggregation (residue range 61–95). On other hand, $\beta$- and $\gamma$-synucleins^8^ helps to inhibit the aggregation process Asyn. The mutations on genomic encoding of SNCA gene have been identified as major element in Lewy bodies. So far 16 loci (PARK1 to PARK16) and 11 genes have been associated with pathogenic progression of PD.

Impacts of such sequential changes are significant. For example, the amyloid formation mechanism of alpha-synuclein is mostly associated with a monomeric primary level of nucleation^9^. Usually the aggregation rate is too slow to detect. However, the phenotypical modifications due to mentioned mutations would have been affecting the residues at NAC domain (which is the prime point of aggregation). Therefore, intra-molecular allosteric effects would lead mutant to behave like ancestral phenotypes. However, these activities are directly affecting the functional profiles of the targeted proteins. Modifications, enhanced due to phenotypical changes, are correlated with the ontology terms. These are shared as common semantics among the pathways separately for wild type (WT) protein their mutants and closest neighbors from each of the cases. During the pathogenic progression, phenotypical modifications can explain the internal orchestration of the pathways based on the mutated candidate and their interacting neighbors. Mostly, these connections are observed for the mutated candidates by following the ancestral protein neighbors. A point mutation at a certain residual position cannot change the whole structure. However, it can affect the co-evoluting residues. Therefore, the relation among pathways is modified which may lead to the initiation of neurodegenerative progression.

In this study, one frame has been proposed by considering the sequential-structural modifications, and pathway semantics networks for Asyn and their mutants. The experiment has been initiated focusing on five mutations from early and late onset PD cases have been A30P, A53T, G51D, E46K, and H50Q. Firstly, the sequential trait of the synuclein family is observed. Then evolutionary conserved co-evolving patches are studied by performing the Direct Coupling Analysis (DCA). Subsequently the information fetched from sequence space has been mapped to the structure. For five mutated cases, two distinct structures has been predicted applying *ab-initio* method. For the wild type protein, the ensemble model has been selected from the known structures. There after, the structure network has been formed based on normal mode scores of each residue. Comparing these information, the effective residues due to mutations have been fetched. Subsequently, BLAST helps to find the sequential similarity with ancestors from the same family. This may provide different lists of neighboring interaction partners for the wild type and mutant. Finally, weighted networks are formed based on pathway semantic relations. The pathways and their relations represent the nodes and the edges of the network. The weight of the edges are computed based on the semantic similarities.

## Results

It is known that $\alpha$-Synuclein is the candidate protein of Parkinson’s disease. In order to understand the effect of this protein and its mutated samples towards the neurodegenerative diseases, a frame is proposed based on sequential and structural changes along with the pathway semantics.

### Sequence analysis

**Figure 1:**
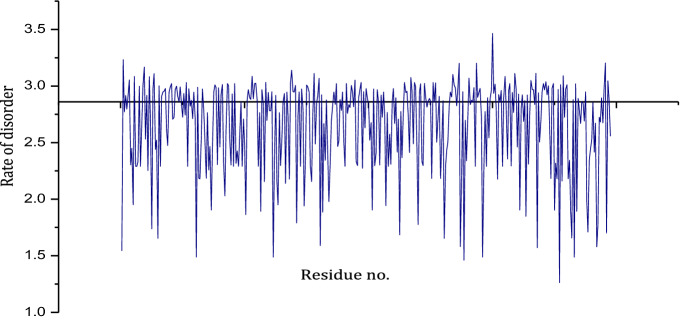
The changing rate of Shannon entropic score of the protein members from synuclein protein family (Pfam id. PF01387).

In sequence space, the family trait of the Synuclein family is analyzed in terms of order and disorder. In this regard, Shannon entropy is calculated for each protein belongs to this family, shown in Fig. 1. Moreover, the entropic score of consensus sequence is 2.58. This suggests that the family trait of Synuclein family is ordered, whereas many proteins belong to this family are disorder in nature. This gives an in-depth idea of evolution.

**Figure 2:**
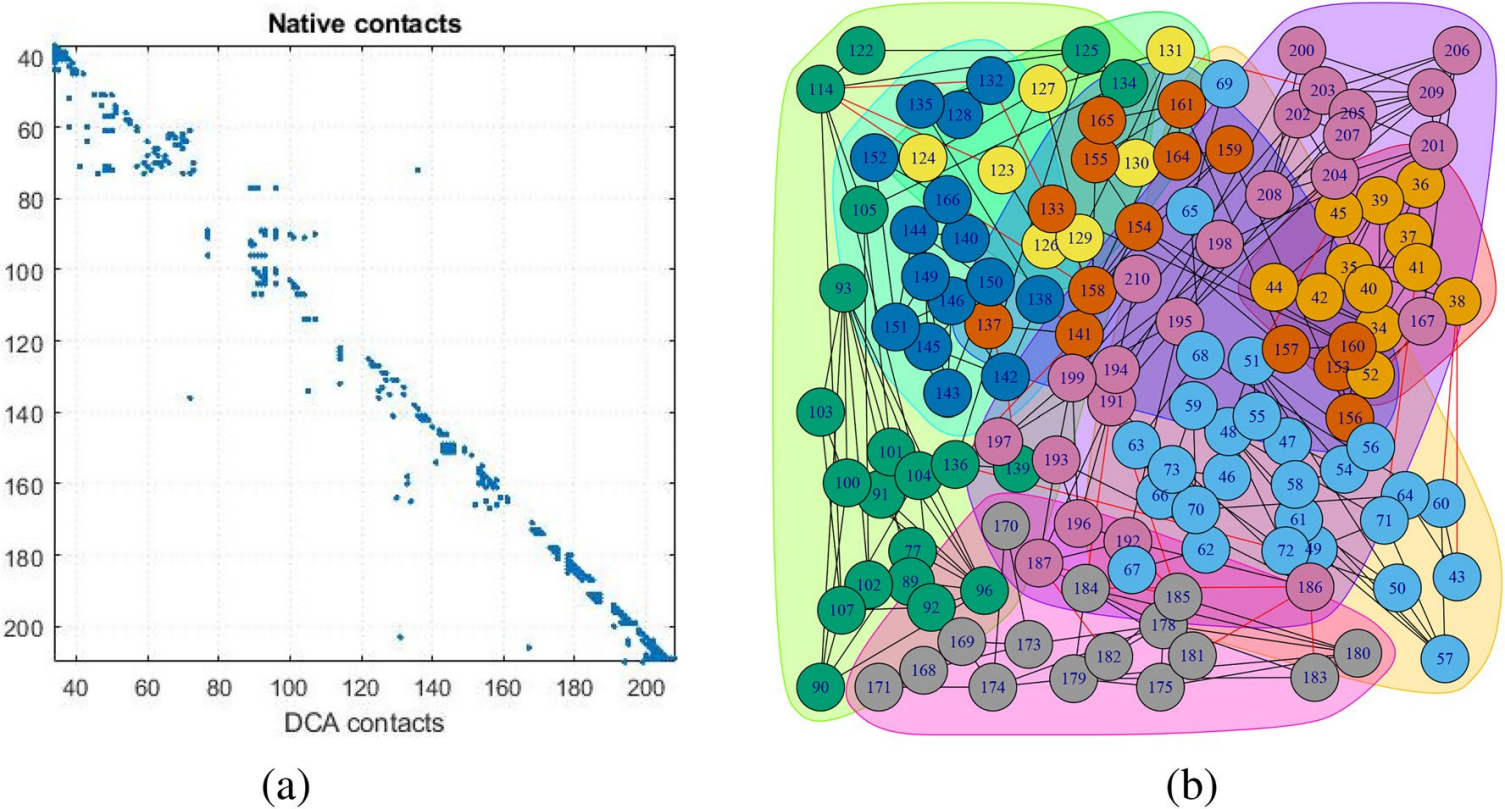
DI scores based on top DCA contacts, (**a**) The co-evolved residue patches at NAC domain. (**b**) A weighted network $G_{DCA}$ and corresponding color modules based on over all residual co-variation from DI score.

To support the evolutionary changes Direct Coupling Analysis (DCA) is performed. The co-varying residue patches are represented in Fig. 2a. DCA score indicates the change in one residue is responsible for the change in other coupled residues. The result depicts that the residue changing for point mutation is also responsible for the change of its co-varying patches which eventually contributes to slow as well as accelerating aggregation of $\alpha$-Synuclein. From the analysis, among five mutations E46K, H50Q and G51D mutated samples show conservation throughout the evolution. In parallel, the residue patches are clustered depending on their DCA score and residue in one cluster denotes same evolution rate. Interestingly, the mutated residues are present in one cluster shown in Fig. 2b, which indicates that the residue present in that cluster are varying accordingly.

### Selection of structure and analysis

**Figure 3:**
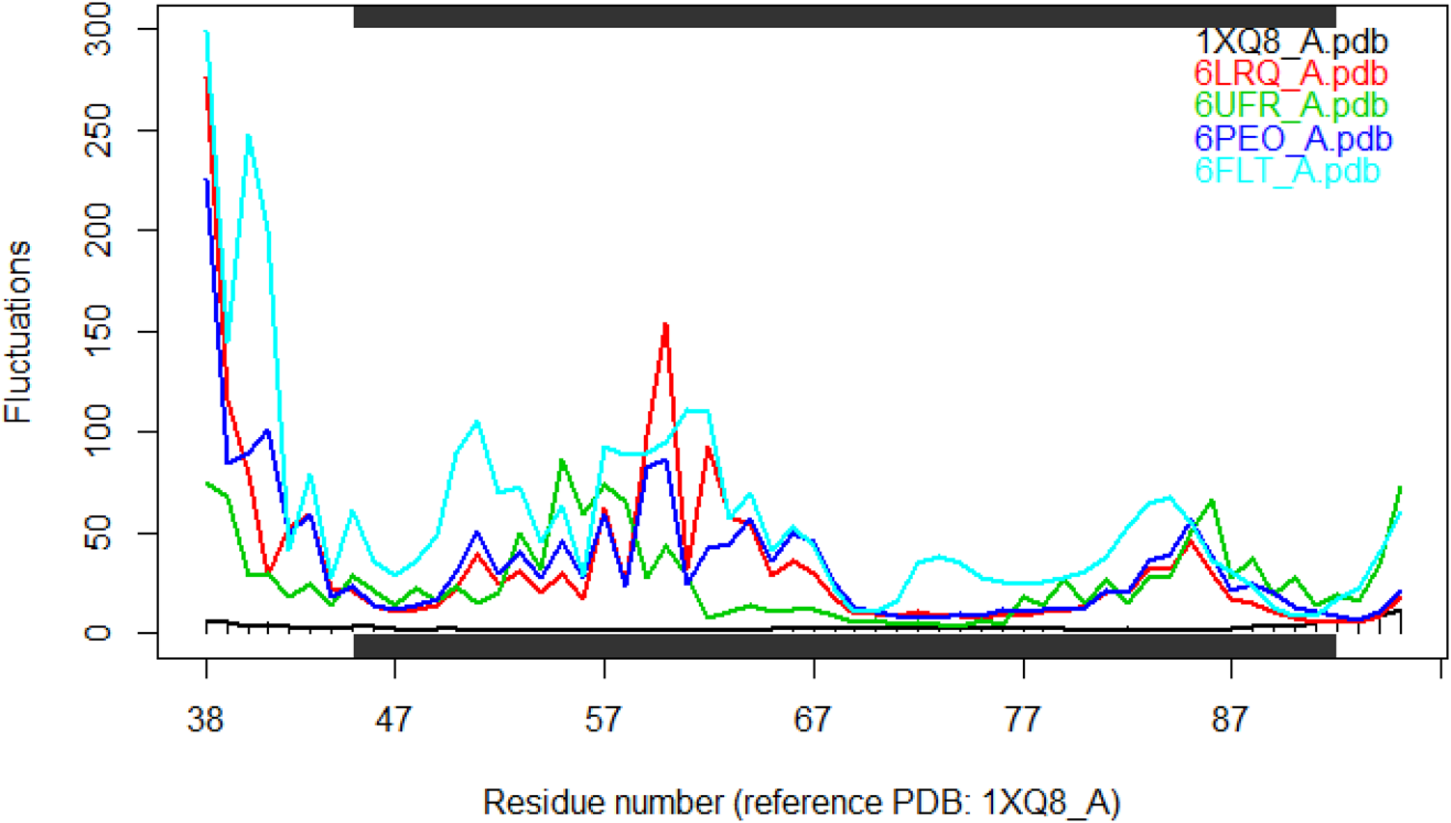
Square fluctuation map on PDB structures of alpha synuclein where each color represents one monomeric chain.

**Figure 4:**
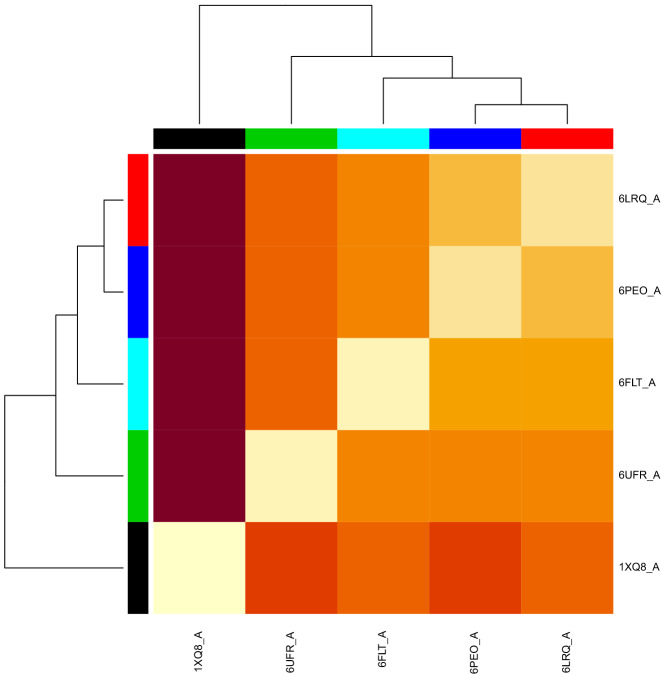
A Heatmap to show structural affinity among the monomeric PDB structures of the Alpha Synuclein where density of color reflected ensemble similarity with other stucture based on Normal Mode score.

$\alpha$-Synuclein has multiple dynamic structure with frequent mutation. Due to this, an ensemble of all the structures is performed to understand the fluctuation rate, shown in Fig. 3. Moreover, a heatmap (Fig. 4) of all the structures also implies the minimum energy and fluctuation rate of structure ID 1XQ8. In this regard, we selected this PDB structure for the further analysis.

In order to have a comprehensive grasp of the changes in sequence space, the PDB structures of $\alpha$-Synuclein and the mutated samples are constructed from the IntFOLD protein prediction server^10^. Based on the PDB models, structure networks are established. The structure network provides a new insight towards the nature and essential factors of structure-function dynamicity and folding process of a protein.

**Figure 5:**
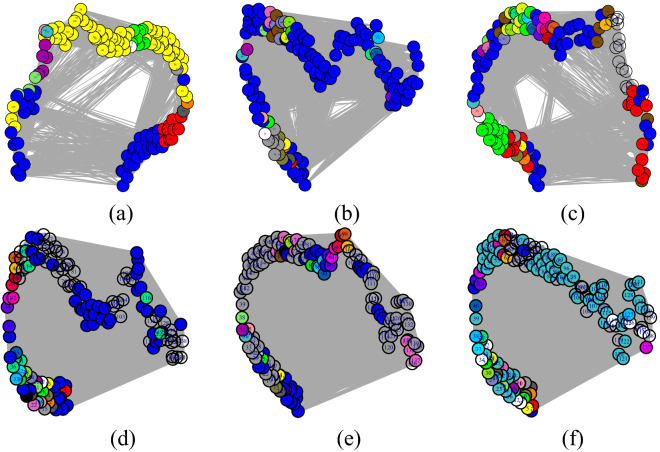
The structure network of (**a**) WT-human, (**b**) G51D-slow, (**c**) E46K-fast, (**d**) H50Q, (**e**) A53T and (**f**) A30P models.

**Figure 6:**
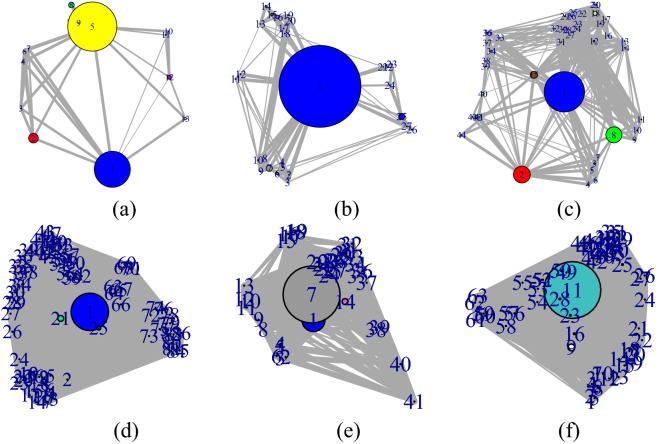
The community cluster modules of structures (**a**) WT-human, (**b**) G51D-slow, (**c**) E46K-fast, (**d**) H50Q, (**e**) A53T and (**f**) A30P after performing modularity detection.

The community detection of a protein is crucial to understand evolution. In this study, every residue pair, obtained from structure network (as shown in Fig. 5a–f) belongs to the same cluster or in a different cluster with a strong association between them as shown in Fig. 6 for 5 mutation along with WT-human. This result also supports the DCA score. Additionally, the betweeness centrality of the mutations are shown in Supplementary Figure F1.

Furthermore, the residue present in modules are compared with cluster residue of Fig. 2b in which the mutated residues are available. The common residues present in both the cluster in the stretch of the NAC domain are mutated accordingly for three models in Fig. 5. The stretch are further consider for performing BLAST. The highest similarity sequence is identified and applied for establishing a PPI network.

The sequence with highest similarity of each sequential models after BLAST are used to understand the interaction with other protein by PPI network. From the BLAST result, it is found that mutation G51D-slow, E46K-fast and H50Q, A53T, A30P have more similarity with Pan paniscus, Pan troglodytes, Sus scrofa, Gorilla gorilla gorilla and Erythrocebus patas respectively. With the cutoff, 0.5 top 11 including SNCA ($\alpha$-Synuclein) are selected. The details list of associated proteins with their scoring depending on the shortest distance from SNCA are listed in Table 1. From this table it is clear that most of the associating proteins are remain common among organisms which depicts that, these proteins remain connected through the evolution of $\alpha$-Synuclein protein. It is concluded that protein may remain to interact but their connection scores change from one species to other.

**Table 1:** The associating proteins of $\alpha$-Synuclein for all the six sequential models are listed.

WT-human		G51D-slow		E46K-fast		H50Q		A53T		A30P	
Annotations	Score	Annotations	Score	Annotations	Score	Annotations	Score	Annotations	Score	Annotations	Score
STUB1	0.996	PINK1	0.933	SLC6A3	0.939	SNCA	0.835	APP	0.741	SNCA	0.908
SLC6A3	0.99	LRRK2	0.861	DYRK1A	0.847	PINK1	0.847	TH	0.785	SLC6A3	0.754
UCHL1	0.985	MAPT	0.84	LRRK2	0.842	PARK7	0.796	DYRK1A	0.763	PINK1	0.82
APP	0.983	APP	0.818	SNCAIP	0.808	PARK2	0.726	PARK7	0.795	PARK7	0.772
LRRK2	0.971	DYRK1A	0.788	SNCA	0.795	MECOM	0.673	PINK1	0.824	PARK2	0.801
HSPA4	0.97	SLC6A3	0.775	MAPT	0.749	MAPT	0.713	SNCAIP	0.92	MAPT	0.658
SNCA	0.969	PARK2	0.725	PINK1	0.724	LRRK2	0.884	MAPT	0.714	LRRK2	0.919
FYN	0.968	SNCAIP	0.717	ATP13A2	0.696	HDH	0.724	CHM	0.664	DYRK1A	0.747
PARK2	0.965	PARK7	0.706	TH	0.696	APP	0.764	SLC6A3	0.726	APP	0.751

### Pathway semantic similarity

The six proteins are further studied in details to understand the role of pathways during evolution. From Reactome^11^ and Enrichr^12,13^ associated pathways of the six proteins are curated. We consider those pathways having more than two proteins including $\alpha$-Synuclein. The biological process and cellular component are considered for each pathway and a semantic similarity graph is constructed shown in Fig. 9. The semantic network of mutation G51D-slow and A30P are shown in Supplementary Figure F2. Each colour of the nodes represent a particular pathway and the edges between them depict the semantic scores. Moreover, the bold edges of the pathway graphs represent the connection weight between two pathways depending on the common biological terms. Additionally, we have included a Table 2 where we have validated few outcomes from literature (rest are shown in Supplementary Table T1). The table helps to conclude that this model provides an anonymous pipeline. There is no such model which targets the desired biological questionnaire best of our knowledge.

**Table 2:** Validating the pathway outcomes from the available literature.

Mutation	Pathway	Interaction partners	Litrature survey
WT-Human	Parkinson’s disease	PARK2,LRRK2, APP	^14–16^
	Dopaminergic	APP, LRRK2	^17,18^
	Alzheimers disease	LRRK2, PARK2	^15,19^
	Monoamine transport	PARK2, SLC6A3	^15^
A53T	Dopaminergic synapse	APP,LRRK2,PARK7,PINK1,SNCAIP	^20–22^
	Amphetamine addiction	PARK7	^23^
	MAPK signaling pathway	APP,LRRK2,PARK7,PINK1	^24–27^
	Parkinson	APP,LRRK2,DYRK1A,PARK7,PINK1,SNCAIP	^2,28–32^
	Alzhimer	APP,LRRK2,DYRK1A,PARK7,PINK1,SNCAIP	^19,32–36^
E46K	Parkinson’s disease	SLC6A3,PINK1,LRRK2	^37,38^
	Alzheimer’s disease	MAPT,PINK1,LRRK2	^37,39,40^
	Cocaine addiction	SLC6A3,TH	^41,42^
	Amphetamine addiction	SLC6A3,TH	^43,44^
H50Q	Parkinson disease	STUB1,SNCAIP,SLC6A3,UCHL1,APP,LRRK2,HSPA4,FYN,PARK2	^36,45–48^
	Alzheimer disease	APP,SNCAIP,UCHL1,LRRK2,FYN	^33,39,45,49^
	MAPK signaling pathway	LRRK2,FYN	^50,51^
	Dopaminergic synapse	LRRK2,SLC6A3	^52,53^
	Mitophagy	SLC6A3,STUB1,SNCAIP,UCHL1,APP,LRRK2,HSPA4	^54–59^

**Figure 7:**
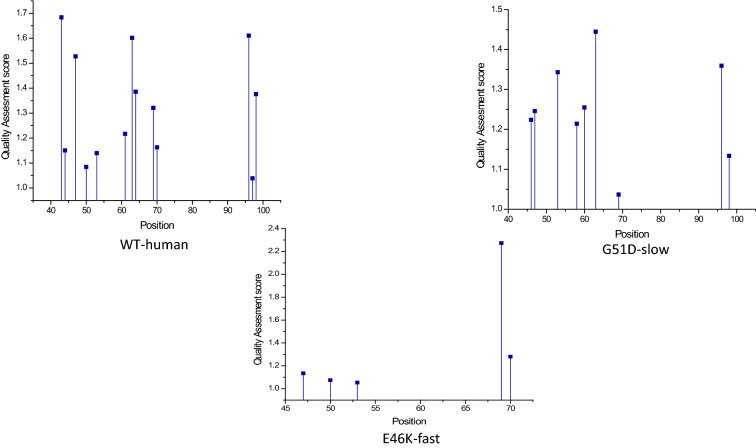
Comparative study on WT-human, G51D-slow and E46K-fast based on variation in Van der Waal’s clashes.

**Figure 8:**
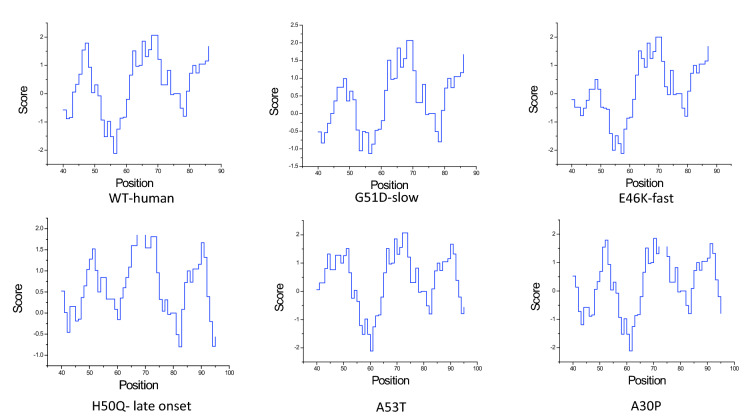
Comparative study on WT-human, G51D-slow, E46K-fast, H50Q, A53T and A30P based on hydropathic Index from 40 to 95 residual points.

In Fig. 7, the certain courses of Van der Waals interaction are shown of the domain 40–95 where mutations mostly occur for three sequential models for he structure network of (a) WT-human, (b) G51D-slow, (c) E46K-fast other three mutations are reported in Supplementary Table T2. Along with the non-covalent force the hydrophobic profiling is represented through a graph in Fig. 8.

An adjacency matrix of each type is obtained during pathway semantic calculation. This matrix is used as input for PR algorithm to rank the pathways according to their importance. From the resulted rank list we noticed that apart from Parkinson disease and Alzhimer’s disease dopaminergic synapse, Amphetamine addiction and Cocaine addiction secured a higher position in all five mutations. However in the WT-type parkin ubiquitin proteasomal system pathway ranked high which is absent in both the mutations. A table of each mutation of alpha-synuclein along with their PR score is reported in Supplementary Table T3.

## Discussion

**Figure 9:**
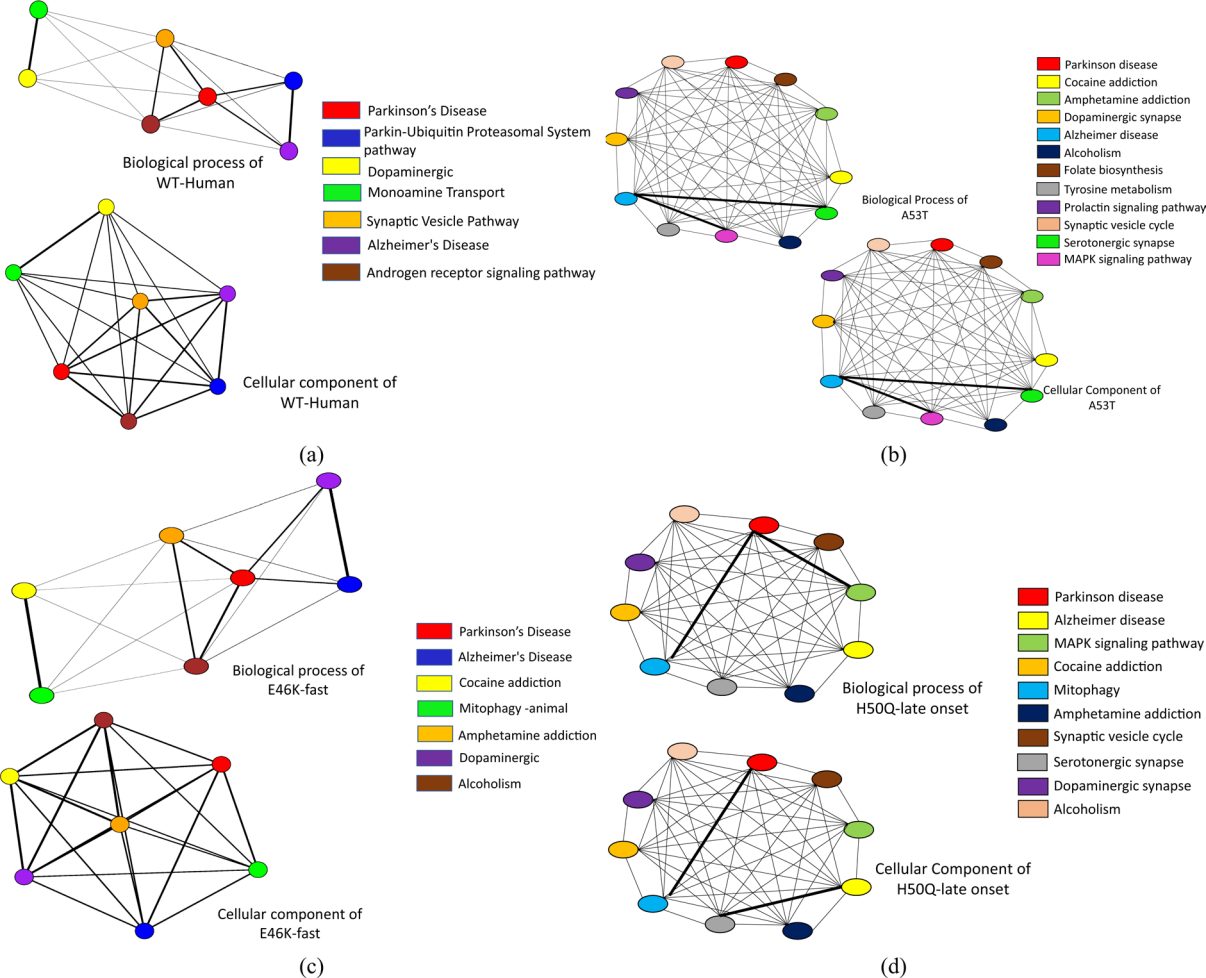
The pathway semantic graph of (**a**) WT-Human, (**b**) A53T, (**c**) E46K-fast and (**d**) H50Q-late onset mutations of Alpha-synuclein. In the graph, the color nodes represent a particular pathway associated with the mutation type and the edges represents the weighted connection between the pathways. The bold edges indicate the higher association between those pathways.

The dynamic nature of the $\alpha$-synuclein lies on the NAC domain. As this domain has a higher propensity of aggregation, it can be considered as the most unstable region. As discussed before, the $\beta$-synuclein and $\gamma$-synuclein proteins are paralogous to $\alpha$-synuclein. However, $\alpha$-synuclein has two more domains. SE scores on each member from the synuclein family indicate that overall trait of the family is highly ordered. In the case of $\alpha$-synuclein, a certain range of disorder has been observed. This trait indicates the unstructured region of the $\alpha$-synuclein. Furthermore, from coupling analysis, 291 potential coupling pairs have been found. Among them, very few have fallen under NAC domain range. However, the mutated samples, namely, A53T, H50Q, G51D and E46K, are not known as part of the aggregation domain. So, the preliminary level of aggregations is somehow influenced by such mutations. Residual association within the protein is majorly controlled by the covalent and non-covalent interactions. In folding purposes, non-covalent forces such as weak van der Waals and ionic interactions play a vital role. Also, the hydrophobic residual shields guard the hydrophilic residues against misfolding. Following this theory, the abruption at non-covalent forces is not allowing the proper folding activity. In Fig. 7, the certain courses of non-covalent interaction have been shown at the affected targeted region at three sequential models. With that, hydrophobic profiling of the models are assist to describe the unstructured nature. In Fig. 8, the individual hydrophobic profiling of the WT and five mutants have been shown. Strikingly, the profiling of WT and E46k-fast are more similar including A30P. H50Q and G51D are also similar. Therefore, the folding pattern of these mutations are supposed to be similar. On other hand, generic extensive effects of the co-evolution at a group of residues have been shown through network $G_{DCA}$ (shown in Fig. 2a). To make a comprehensive understanding, the sequence space information is mapped to individual structural information corresponding to the structure networks WT, G51D-slow and E46K-fast (shown in Fig. 5). From the colour modules of the $G_{NMA}$, it is observed that the distribution of the clusters is giving proper evidence of the aggregation. As each networks has the same tree cutter threshold, the distribution of the modules should be unbiased. Following this trait, it has been observed that G51D-slow has a higher propensity of aggregation than the other five. Mohite et al.^60^ shows the strong effect of G51D phenotypical changes. In the rest of mutation, structure network can not provide much relevant information. All these monomeric models are highly prone to aggregation that reflect in the normal mode based weighted networks. Most of the residue nodes are conserved within fewer communities. From Fig. 2a, the coupling propensity of the NAC domain has shown. Interestingly, the distribution of co-evolutionary patches of the NAC domain is large. Comparing with individual structure networks, the individual lists of the affected residues due to an individual set of mutations are considered to be found the ancestral sequential traits. Eventually, the sequence traits are indicating the type of structural aberration from the WT. Subsequently, structural modifications in each case can provide a different list of top interaction partners. In Table 1, the list of interaction partners for the individual types are given. Few of the pathways are usually performed in every eukaryotic cell. However, the activities of the pathways are revised based on their metabolome. In Fig. 9, networks based on semantic similarity have been shown. From the networks, all the non-pathogenic pathways associated mostly with the common top neighbors of $\alpha$-synuclein (with few protein sample specific neighbors) are semantically associated with each other. BP has been considered for the similarity calculation. In WT-human, the non-pathogenic pathways have almost homogeneous association where the connection between blue and yellow node is slightly strong. In E46K-fast, this association between blue and yellow node is extremely strong. This information is comparable with the module distribution of the $G_{NMA}$ where the propensity of aggregation is highly strong in G51D-slow.

The activities of Parkin-Ubiquitin Proteasomal System pathway and Ectoderm Differentiation are highly influential in the activity of the Dopaminergic Neurons which is key point of PD initiations^61,62^. However, strong association between the selected pathways has shown in semantic networks. From the pathway list, it has been observed that few pathways viz., Mitophagy, Dopaminergic synapses, MAPK signaling pathway, Amphetamine addiction are available in almost all prime mutations, and WT type alpha-synuclein. As per previous evidence, the association between the dopaminergic synapses and mitophagy can be explained. In^63^, the association of mitochondrial dysfunction and PD has been discussed. Dopaminergic Neurons are one of the highest consumers of ATP which explains the contribution of mitochondrial dysfunction in neuronal death^64^. Two perspectives so far can explain the relation clearly- firstly, the presence of the unpaired electrons accelerates ROS. ROS elevation facilitates aging by activating antioxidant enzymes as well as transporters. Subsequently, this also helps to relocate dopamine from the intercellular medium to synaptic vesicles. This whole process is associated with cell death where ROS levels increase throughout tissues including the brain for affected patients. Likewise, mitophagy involves the maintenance of a highly interconnected network throughout neurons. Balancing the mitochondrial activity in healthy cases needs the fusion and fission process^65^. Any sort of disruption main leads to aggregation and loss of direct movement. The common key between this activity is the mutations of mtDNA^66^ which disrupts the complex I and stops the mitophagy process. Interestingly, early-onset A53T mutation also follows the same path which leads to PD^67^. As per our results, most of the mutations are associated with mitophagy and dopaminergic synapses. Therefore, we are expecting that the mutations may have followed a similar path through mitochondrial dysfunction. However, two early-onset mutations i.e., A53T and A30P are observed to be involved in the MAPK signaling pathway. This explains the alternative path through MAPK kinase^68^. The dysfunction of such kinase leads during oxidative stress can responsible for mitochondrial stress and lead to mitochondrial dysfunction. Similarly, upregulating E46k is directly associated with the autophagy mechanism which controls the mitochondrial fission^69^. As per our study, some of the nodes from the pathway semantic networks (shown in Fig. 9) can be explained through previous researches. The other nodes from each network are expected to be partially associated with the regulation of the influential nodes. In Table, results have been validated through existing literature where we focused on the associated interaction partners of the mutants. Hence, the semantic networks can be considered as a summarization of the mutant specific molecular mechanism.

Aging is one of the prime reasons for losing the cognitive senses. WT type Asyn is usually coming up with cellular precipitation which leads to dementia. However, the early onset mutation can trigger the precipitation within 30–40 years of the age range. Mutational subtypes i.e., A30P, A53T, G51D-slow and E46K-fast, are studied to unveil the functional and molecular orchestration during such mutations. The associated list of pathways individually for these samples has been considered. The pathways are connected through the sharing of interacting neighbors. Here, the list of protein interacting neighbors is modifying with mutations which brings us the distinct list of pathways as well as semantic connectivity. Twelve networks are based on two cognitive parameters i.e., Cellular component and, Biological process. From the networks, all the associated pathways are clearly sharing almost the same subcellular localization. In WT type, the pathways excluding Parkinson’s Disease and Alzheimer’s Disease are associated with two distinct types of the function where some pathways are associated with prevention from unusual activity within the neurons whereas the rest of the pathways are associated with the pathogenic progression of the diseases. For example, monoamine transport systems are neurotransmitters that prevent the extracellular vesicles of the neuronal cells from excess dopamine, serotonin, noradrenaline, etc. Dopaminergic pathway, found in the list, is one of the prime pathways from the neuronal cells which helps to control the cognitive senses. Monoamine transport systems are the key modulator of the dopaminergic pathways. It can also control the effects of nanomolar elements such as cocaine, amphetamine, etc. On the other hand, pathways like Parkin-Ubiquitin Proteasomal System regulates the misfolding of the proteins which are further responsible for the pathogenic progression of the diseases such as PD. The pathway semantic networks (shown in Fig. 9) show the connectivity within the pathways based on sharing list of biological processes and cellular components. In the BP based network, the Parkin-Ubiquitin Proteasomal System pathway is the highest-ranked pathway as per PR outcomes. This shows the influence of the pathway in the network which increases the possibility of the Asyn misfolding.

The pathways of the selected mutations Viz., G51D-slow and E46K-fast, are mostly associated with external nanomolar elements. More elaborately, dopaminergic pathways are strongly associated with the proteins which are differentially regulated due to cocaine addiction, alcoholism, amphetamine addiction^70–72^. Amphetamine, cocaine are known neurotransmitters. Although appropriate etiology associated with amphetamine is not known, amphetamines are initially used to treat PD^73^. However, some recent studies have shown that long use of amphetamine-type stimulators increases the risk of PD. The studies explain that amphetamine-type stimulators bind in the intrinsically disordered regions of the Asyn protein and facilitates the molecular mechanism of the aggregation. The studies also suggest that it promotes the post-translational modification of the Asyn directly or indirectly. Interestingly, the involvement of the Asyn mutants specially, H50Q and G51D have largely been observed in PD cases who are long users of such stimulators^74,75^. The decreasing number of dopaminergic neurons at Substantial nigra is also considered as one of the key reasons^76^. Also, the mutations can inhibit the activities of the neurotransmitters such as monoamine transport systems^77^. Due to that reason, the excess hormones in the cellular systems cannot be removed. As per the pathway semantic networks, E46K-fast shows the aforementioned strong connectivity. However, the amphetamine addiction pathway is disconnected in terms of BP for G51D-slow.

We have included three more mutations to explain the pd etiology clearly. From the pathway list, it has been observed that few pathways viz., Mitophagy, Dopaminergic synapses, MAPK signaling pathway, Amphetamine addiction are available in almost all prime mutations, and WT type alpha-synuclein. As per previous evidence, the association between the dopaminergic synapses and mitophagy can be explained. In^63^, the association of mitochondrial dysfunction and PD has been discussed. Dopaminergic Neurons are one of the highest consumers of ATP which explains the contribution of mitochondrial dysfunction in neuronal death^64^. Two perspectives so far can explain the relation clearly–firstly, the presence of the unpaired electrons accelerates ROS. ROS elevation facilitates aging by activating antioxidant enzymes as well as transporters. Subsequently, this also helps to relocate dopamine from the intercellular medium to synaptic vesicles. This whole process is associated with cell death where ROS levels increase throughout tissues including the brain for affected patients. Likewise, mitophagy involves the maintenance of a highly interconnected network throughout neurons. Balancing the mitochondrial activity in healthy cases needs the fusion and fission process^65^. Any sort of disruption main leads to aggregation and loss of direct movement. The common key between this activity is the mutations of mtDNA^66^ which disrupts the complex I and stops the mitophagy process. Interestingly, early-onset A53T mutation also follows the same path which leads to PD^67^. As per our results, most of the mutations are associated with mitophagy and dopaminergic synapses. Therefore, we are expecting that the mutations may have followed a similar path through mitochondrial dysfunction. However, two early-onset mutations i.e., A53T and A30P are observed to be involved in the MAPK signaling pathway. This explains the alternative path through MAPK kinase^68^. The dysfunction of such kinase leads during oxidative stress can responsible for mitochondrial stress and lead to mitochondrial dysfunction. Similarly, upregulating E46k is directly associated with the autophagy mechanism which controls the mitochondrial fission^69^.

Outcomes of the PR algorithm shows affinity of all the mutations with Amphetamine addictions, Cocaine addictions, dopaminergic synapses, etc. Also, H50Q and A30P have synaptic vesicles among the top ranking pathways. These observation supports the etiology associated with mitochondrial dysfunction. Interestingly, few pathways which are mostly associated with dopaminergic disorientation, are identified under A53T mutation.

## Conclusion

From this study, the sequential variation of $\alpha$-synuclein is observed depending on six mutational conditions, considering the structural consequences. The objective of the study is also to observe changes in pathways due to structural aberration. Among the mutations, the pathway semantic networks of A53T and G51D show maximum involvement of pathways. Also, few pathways play vital roles in mutational perspectives viz., Amyloid fiber formation, Parkin-Ubiquitin Proteasomal System pathway, Ectoderm Differentiation, Mitophagy, MAPK signaling pathway, synaptic vesicles, etc. Finally, the proposed frame can provide a comprehensive outlook on the mutation mediated structural aberrations and their affects on functional pathways.

## Method

**Figure 10:**
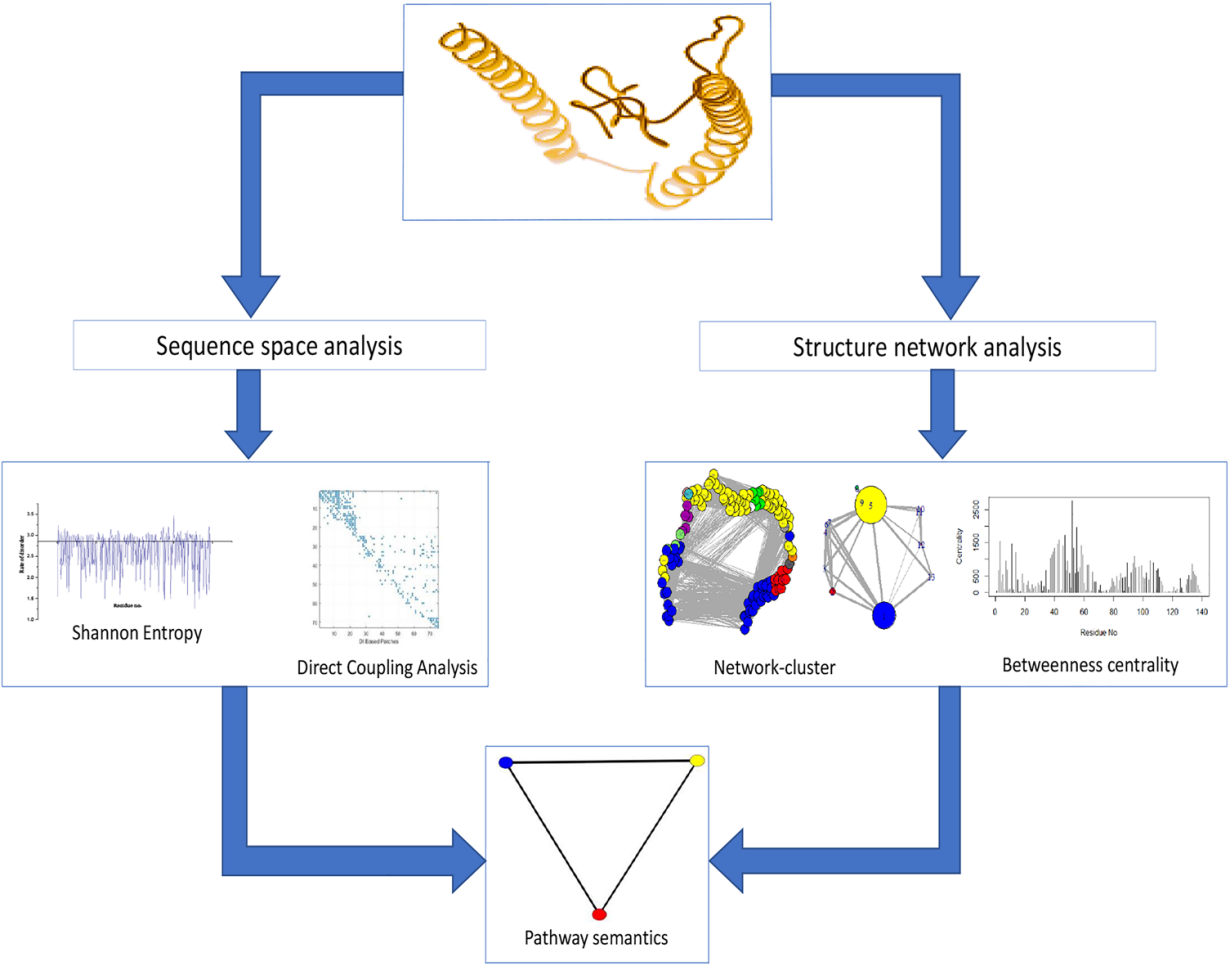
The flowchart of the proposed framework.

This study aims to understand the structural changes along with the pathway semantics of $\alpha$-Synuclein concerning evolution. The detailed method is described in Fig. 10.

### Sequence space analysis

Primarily, the Synuclein family is considered to unveil the family trait with the help of Shannon entropy (SE) calculation. It is evident that Shannon entropy is directly proportional to the rate of disorder. This implicates if the entropic score increases it signifies the higher disorderness of a protein. In^78^, 2.9 is considered as the threshold of Shannon entropy score, i.e. sequence with score less than 2.9 is treated as ordered sequence and vice-versa. This idea is utilized in this study and applied on each protein sequences of Synuclein family. Shannon entropy is calculated as follows:

1 $$\begin{aligned} Shannon_{E}(i)=-\sum _{i=1}^{L}H_i log_2 H_i \end{aligned}$$

where $H_i$ was the probability of given amino acids and L was the number of letters in a sequence. The summation run over the 20 residues that normally were present in a protein sequence. The probability $H_i$ represent the composition of the consensus sequence. So the entropy range lied between 0 and the $log_2(20) = 4.32$.

Moreover, multiple sequence alignment is performed of the protein sequences belong to PF01387. It is noticed that multiple sequence alignment have a significant role in the field of structure and function analysis of biological sequences. Due to this, full alignment FASTA format sequences were collected, which comprises of tree orderings and all lower case letters including dashes to indicate the gaps. Additionally, a consensus sequence is generated from the aligned sequences. The consensus sequence is a set of amino acids with their occurrence frequency. These frequencies indicate the signature amino acids remain conserved throughout the evolution. In this regard, Shannon entropy is calculated for the consensus sequence also.

The aligned sequences are further considered for DCA. This is a statistical framework which possesses the idea of direct co-evolution coupling among the residue pairs. The drawback of the Mutual Information (MI) is, unable to extricate direct correlation from indirect ones. This problem is easily solved by Direct Information (DI) theory. Depending on the DI theory, DCA is calculated. Hence, the computation of the DCA score of an aligned sequence indicates how directly the selected coinciding residues are coupled with each other and contributed toward evolution. The DI score is defined as:

2 $$\begin{aligned} DI_{s,t}=\sum XX'* F_{s,t}^{{(dir)}}*(X,X') * ln\frac{F_{s,t}^{(dir)}}{F_{s}(X)F_{t}(X')} \end{aligned}$$

Here, $F_{st}^{(dir)}$ represents reweighted frequency counts to introduce two residues for DI. where $F(X,X')$ is considered as joint probability, and *F*(*X*) and $F(X')$ are individual probability. $F_{s}(X)$ and $F_{t}(X')$ are for amino acid type A at $s$th position and similarly B at $t$th position.

The cascading effect of the co-evolution is not conserved in a couple of residues. It is highly possible that mutational changes at a residual point can affect a distant residue by means of a cascading effect. To understand such an effect, a weighted network $G_{DCA}$ has been defined considering DI score between residue. The weighted undirected network $G_{DCA}$ = ($V_{res}$, $E_{DI}$) where $V_{res}$ are set of nodes, consist of residue whereas $E_{DI}$ are weighted edges, consists of DI score between the residues. Subsequently, the color modules have been formed applying Girvan-Newman algorithm^79^.

#### Root mean square fluctuation

The information related to sequence space or structure space individually is not enough to understand the changes. For the study, the WT type protein is taken from Protein Data Bank where the respective PDB id is 1XQ8. Due to the dynamic structure and frequent mutations, the structure possess lowest energy is considered. In this regard, Root Mean Square Fluctuation (RMSF) is performed to measure the particle deviation. In RMSF, a mean over time is considered for a residue *r* at the current position and some reference position. The definition of the RMSF is given in Equation 4.

3 $$\begin{aligned} RSMF_r=\bigg (\frac{1}{S}\sum \limits _{t_n=1}^{S}mod(R_i(t_n)-R_i^{re})^2 \bigg )^{.05} \end{aligned}$$

Where *S* is time over which the mean has been taken for reference position of the particle *i* , $R_i^{re}$. The RMSF has been observed based on the reference position of the particle *i* over time.0

### Structure network analysis

To grasp this observation we have built the structure of those sequences. These complex models are analyzed with the help of a structure network^80^. The interaction between the elements of the networks is represented through nodes and edges. Generally, a secondary structure and folding arrangement mechanism are used to understand the structure of a protein. Another promising methodology for understanding the structure is through network. The equation is represented below.

4 $$\begin{aligned} E_{ab}= \left[\frac{sc_{ab}}{\sqrt{(sc_a*sc_b)}} \right]*100 \ge E_c \end{aligned}$$

$E_c$ is the threshold of interaction strength, the default value is 4%. Here, $sc_{ab}$ was the number of side chain atom pairs of residues *a* and *b*. $sc_a$ and $X_b$ were the normalization factor for residues types *a* and *b*^81^.

In this paper, depending on the normal mode analysis (NMA) a correlation matrix is obtained. The matrix is applied to establish a full residue weighted network $G_{NMA}$ = ($V_{res}$, $E_{NM}$) where $V_{res}$ is a set of nodes representing residues whereas $E_{NM}$ is set of weighted edges where weights are obtained from correlation matrix. Consequently, the network is split into a highly correlated coarse-grained community cluster network by using Girvan-Newman^79^ clustering method where the highly interacting residues were clumped together in the clusters with a threshold value 0.7.

### Pathway semantic

Furthermore, mutation G51D and E46K i.e., slow and accelerating mutation on early onset are considered for further analysis. During mutation, co-varying residue cluster according to the DCA score are compared with the structure network module. The residues common in both the cases and also belong with the residue number 51 and 46 are mutated accordingly. As it is known that residue range 61–95 is the NAC domain of $\alpha$-Synuclein, the residue stretch from 40 to 95 is utilized for performing Basic Local Alignment Tool (BLAST). The protein sequences are matched with the sequence database and statistical significance is calculated for the matched areas. The highest similarity sequence is selected and Protein-Protein Interaction (PPI)^82^ is executed to detect the associated proteins. PPI is carried out for $\alpha$-Synuclein of three species with a threshold value of 0.5. Pathways responsible for at least two proteins including $\alpha$-Synuclein are considered. Here the semantic similarity is calculated over a set of a biological process responsible for the selected pathways. Wang method^83^ is applied to evaluate the similarity established on graph-like structure of gene ontology (GO). The aggregated contribution is done by the semantic value of GO term *T* to the terms in $DAG_T$ which is semantic of GO term *T* is firstly defined in Wang method. GO terms closer to *T* in $DAG_T$ implies more contribution toward it semantics. Hence, it is defined that the contribution of GO term *p* to the semantics of *T* as the $S{\text{-}}value$ of GO term *p* related to GO term *T*. For whatever term of *p* in $DAG_T$, the $S{\text{-}}value$ associated GO term *T*, $S_T(p)$ is calculated as:

5 $$\begin{aligned} \Bigg \{ \begin{array}{l} S_T(T)=1 \\ S_T(p)=max{(c_e*S_T(p')\mid p' \in children\ of\ (p))}\ if\ p\ne T\\ \end{array} \end{aligned}$$

Here $C_e$ is defined as semantic contribution factor for the edge $e \in E_T$ linking GO term *p* with its child term $p'$. After calculating the $S{\text{-}}value$ for the GO term in $DAG_T$, the semantic value of GO term T, *SV*(*T*) is defined as:

6 $$\begin{aligned} SV(T)=\sum _{p \in X_T} S_T(p) \end{aligned}$$

For two given GO term such as *T* and *Q*, semantic similarity is calculated between them is as follows:

7 $$\begin{aligned} SS_w (T,Q)=\frac{\sum _{p \in X_T \cap X_Q} S_T(p) + S_Q(p) }{SV(T)+SV(Q)} \end{aligned}$$

Here $S_T(P)$ is the $S{\text{-}}value$ of GO term t related to term *T* and $S_Q(p)$ is the $S{\text{-}}value$ of GO term *p* related to term *Q*.

Moreover, based on the semantic similarity of GO terms, Best-Match Average (BMC)^84^ strategy is performed to compute semantic similarity among sets of GO terms associated with the protein associated with a particular pathway and column, which defined as:

8 $$\begin{aligned} S_{BMA}(G1,G2)= \frac{\sum _{1=m}^{i} \max \limits _{1\le n\le j} S(go1_m,go2_n)+\sum _{1=n}^{j}{\max \limits _{1\le m\le i} S(go1_m,go2_n)}}{i+j} \end{aligned}$$

where gene G1 annotated by GO terms sets $GO1=(go_{11},go_{12} \ldots go_{1i})$ and G2 annotated by $GO2=(go_{21},go22 \ldots go_{2j})$.

### Ranking the pathways

The pathway semantic similarity graphs reveal the semantic strength between two pathway. In order to understand the importance of the pathways PageRank (PR)^85^ Algorithm introduced by Google is applied on the resulted networks. This algorithm used probability distribution based on the weight among different nodes. In following equation 9, pathways are represented as nodes and the edges are weighted. The node rank has been defined as:

9 $$\begin{aligned} PR(n) = \sum _{m\in A_{n}}{\frac{PR(m)}{E(m)}} \end{aligned}$$

where the rank of node *n* is relied on the *PR* values for each connected node m $\in$ A$_{n}$, divided by *E*(*m*), edges from node n. Therefore, according to the the *PR* values the pathways are ranked based on their importance in the network.

## Supplementary Information

Supplementary Information 1.

Supplementary Information 2.

Supplementary Information 3.

Supplementary Information 4.

Supplementary Information 5.
