# Supplementary figures and images for: Studying the effect of alpha-synuclein and Parkinson’s disease linked mutants on inter pathway connectivities
Source: Sci Rep. 2021 Aug 11;11:16365. doi: 10.1038/s41598-021-95889-5 (PMC8358055; doi:10.1038/s41598-021-95889-5)

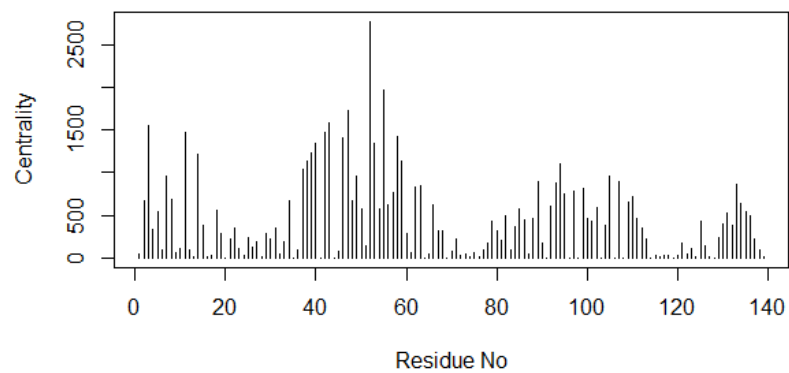

WT-Human

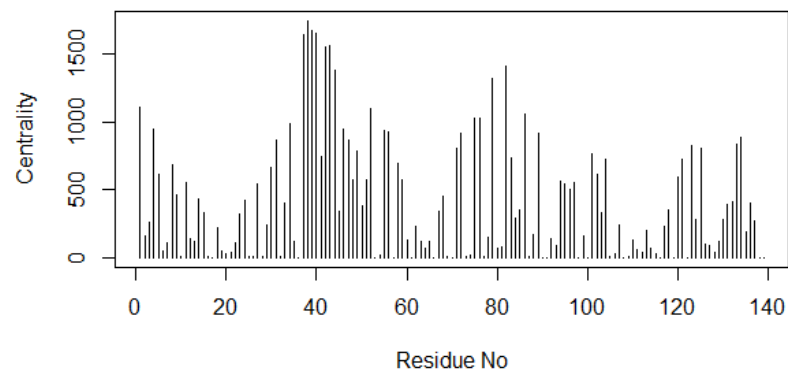

G51D

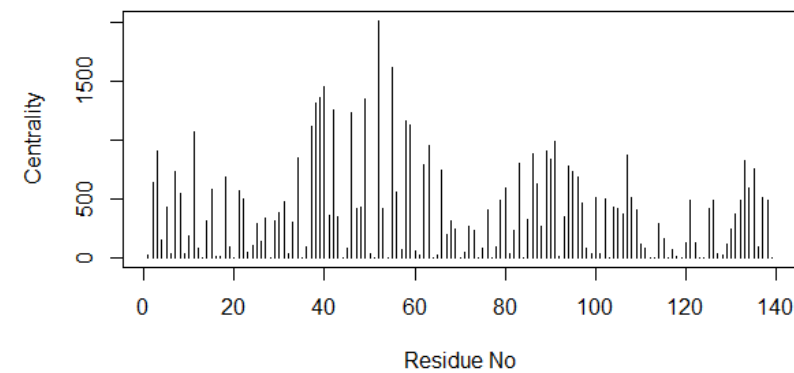

E46K

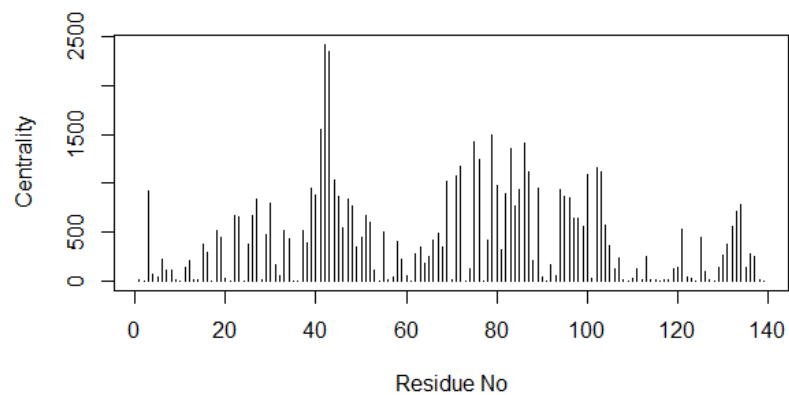

H50Q

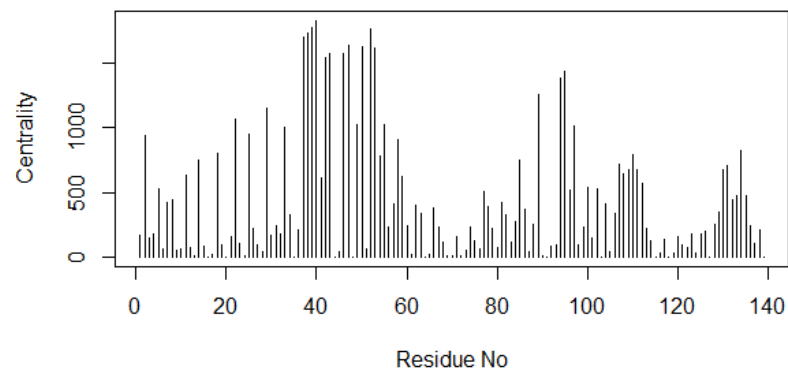

A53T

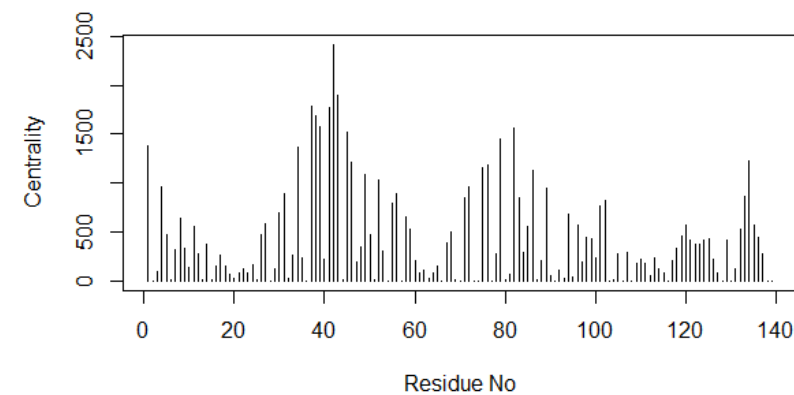

A30P

Supplementary Figure F1. The betweenness centrality of the mutations

Supplement: Supplementary file 1 — Supplementary Information 1. [file 41598_2021_95889_MOESM1_ESM.pdf]
