# Supplementary material for: Studying the effect of alpha-synuclein and Parkinson’s disease linked mutants on inter pathway connectivities
Source: Sci Rep. 2021 Aug 11;11:16365. doi: 10.1038/s41598-021-95889-5 (PMC8358055; doi:10.1038/s41598-021-95889-5)
Supplement: Supplementary file 2 — Supplementary Information 2. [file 41598_2021_95889_MOESM2_ESM.pdf]

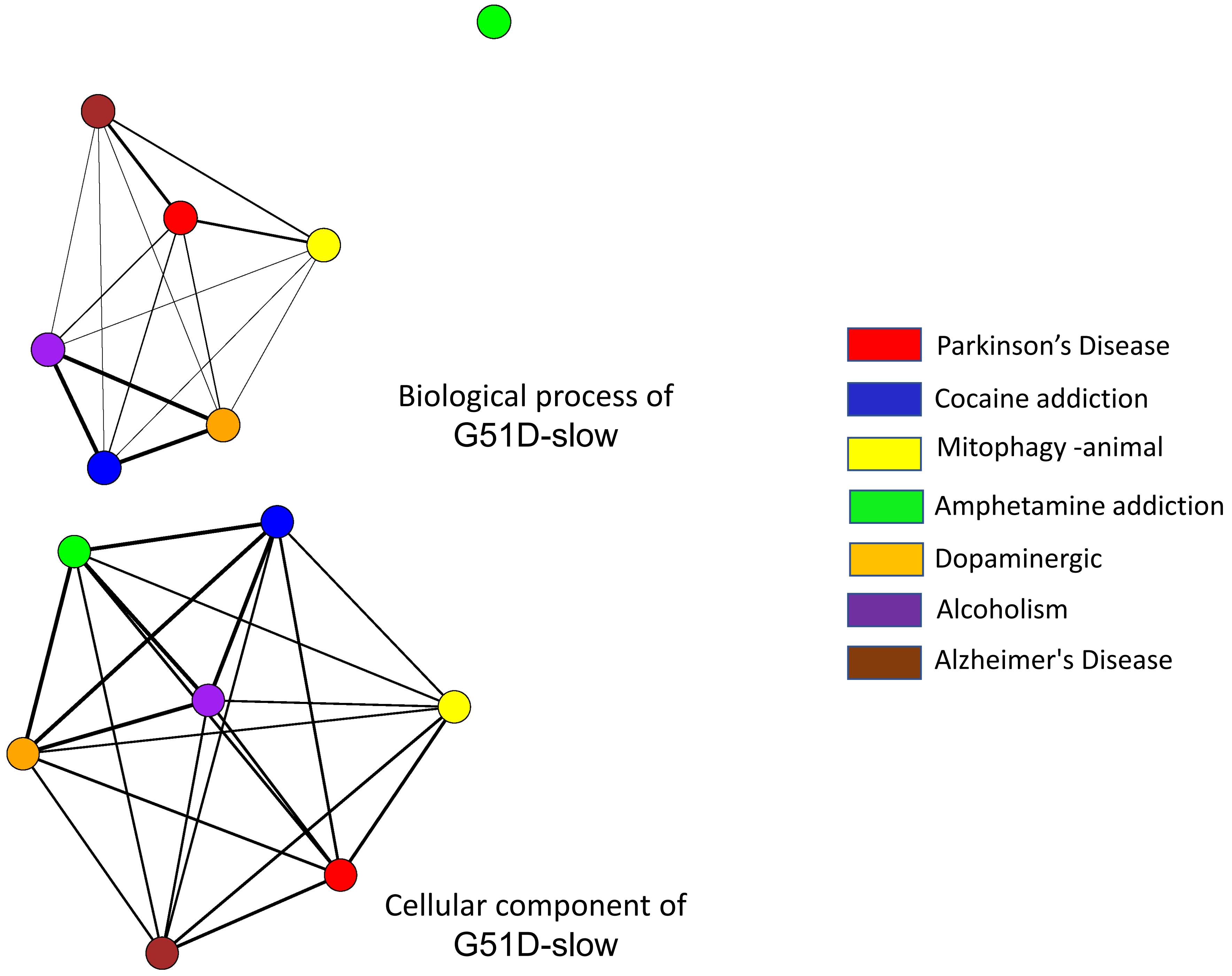
Biological process of
G51D-slow
Cellular component of
G51D-slow
Parkinson’s Disease
Cocaine addiction
Mitophagy -animal
Amphetamine addiction
Dopaminergic
Alcoholism
Alzheimer's Disease

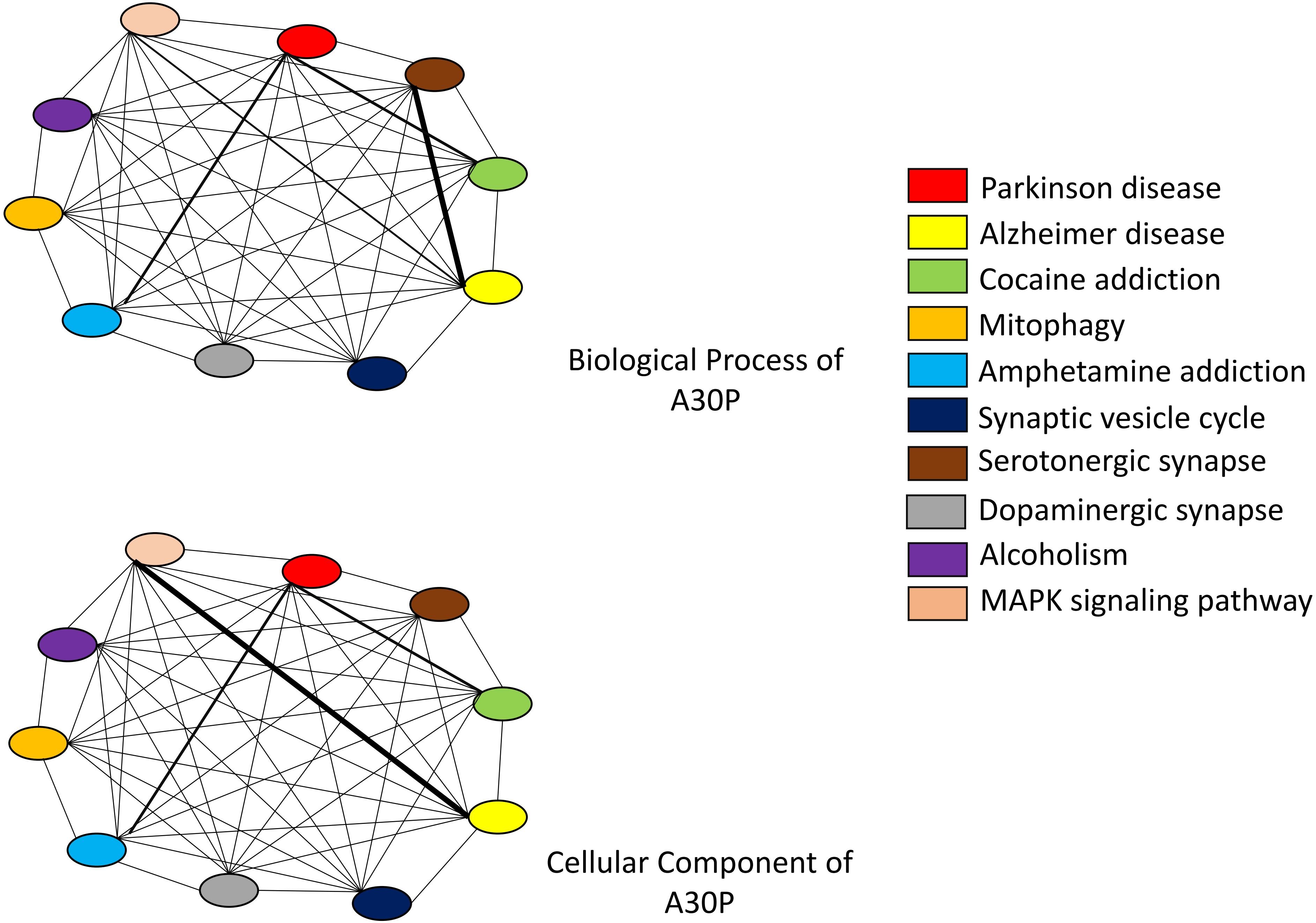

Figure F2. The pathway semantic graph of G51D-slow and A30P mutations of Alpha-synuclein. In the graph, the color nodes represent a particular pathway associated with the mutation type and the edges represents the weighted connection between the pathways. The bold edges indicate the higher association between those pathways.
