# Supplementary material for: Studying the effect of alpha-synuclein and Parkinson’s disease linked mutants on inter pathway connectivities
Source: Sci Rep. 2021 Aug 11;11:16365. doi: 10.1038/s41598-021-95889-5 (PMC8358055; doi:10.1038/s41598-021-95889-5)
Supplement: Supplementary file 3 — Supplementary Information 3. [file 41598_2021_95889_MOESM3_ESM.pdf]

Mutation	Pathway	Interaction partners	litrature survey
G51D	Parkinson's	PINK1,LRRK2,PARK7, PARK2	https://molecularneurodegenerat
			https://www.sciencedirect.com/sc
			https://link.springer.com/article/1
	Alzheimer's	MAPT,PARK7,APP,PINK1	https://www.ncbi.nlm.nih.gov/pm
			https://www.frontiersin.org/articl
			https://www.ncbi.nlm.nih.gov/pm
			https://molecularneurodegenerat
	Dopaminer	PINK1,MAPT,LRRK2	https://www.frontiersin.org/articl
			https://molecularneurodegenerat
			https://www.frontiersin.org/articl
A30P	Parkinson d	SLC6A3,PARK7, PINK1, PARK2, LRRK2,MAPT	https://pubmed.ncbi.nlm.nih.gov/
			https://www.ncbi.nlm.nih.gov/pm
			https://www.ncbi.nlm.nih.gov/pm
	Alzheimer d	APP,MAPT,PINK1,PARK7,PARK2,LRRK2	https://pubmed.ncbi.nlm.nih.gov/
			https://www.ncbi.nlm.nih.gov/pm
			https://www.ncbi.nlm.nih.gov/pm
	Mitophagy	PINK1, MAPT, LRRK2, PARK7, PARK2	https://pubmed.ncbi.nlm.nih.gov/
			https://www.biorxiv.org/content/
			https://pubmed.ncbi.nlm.nih.gov/
	Dopaminer	PINK1	https://pubmed.ncbi.nlm.nih.gov/
	MAPK signa	MAPT, LRRK2,PARK7, PARK2	https://pubmed.ncbi.nlm.nih.gov/

ion.biomedcentral.com/articles/10.1186/s13024-019-0329-1

ion.biomedcentral.com/articles/10.1186/s13024-019-0329-1

ion.biomedcentral.com/articles/10.1186/s13024-015-0038-3

T1. Validating the pathway outcomes from the available literature
