# Supplementary material for: Studying the effect of alpha-synuclein and Parkinson’s disease linked mutants on inter pathway connectivities
Source: Sci Rep. 2021 Aug 11;11:16365. doi: 10.1038/s41598-021-95889-5 (PMC8358055; doi:10.1038/s41598-021-95889-5)
Supplement: Supplementary file 4 — Supplementary Information 4. [file 41598_2021_95889_MOESM4_ESM.pdf]

GLY	40	to ALA energy chang	1.14658
SER	41	to ALA energy chang	-0.14139
LYS	42	to ALA energy chang	-0.53129
THR	43	to ALA energy chang	-1.03643
LYS	44	to ALA energy chang	-0.39261
GLU	45	to ALA energy chang	-0.42406
GLY	46	to ALA energy chang	-0.83016
VAL	47	to ALA energy chang	-0.06115
VAL	48	to ALA energy chang	-0.16566
GLN	49	to ALA energy chang	-0.36162
GLY	50	to ALA energy chang	-0.75262
VAL	51	to ALA energy chang	-0.12837
ALA	52	to ALA energy chang	0
THR	53	to ALA energy chang	-0.65707
VAL	54	to ALA energy chang	-0.40557
ALA	55	to ALA energy chang	0
GLU	56	to ALA energy chang	-1.50207
LYS	57	to ALA energy chang	-1.27338
THR	58	to ALA energy chang	-1.08418
LYS	59	to ALA energy chang	-2.40311
GLU	60	to ALA energy chang	-1.04386
GLN	61	to ALA energy chang	-1.33143
VAL	62	to ALA energy chang	-0.26281
THR	63	to ALA energy chang	-0.20578
ASN	64	to ALA energy chang	-0.16752
VAL	65	to ALA energy chang	-0.3107
GLY	66	to ALA energy chang	0.165118
GLY	67	to ALA energy chang	-0.57038
ALA	68	to ALA energy chang	0
VAL	69	to ALA energy chang	-0.11272
VAL	70	to ALA energy chang	-0.17
THR	71	to ALA energy chang	0.027999
GLY	72	to ALA energy chang	-1.05967
VAL	73	to ALA energy chang	-0.03235
THR	74	to ALA energy chang	-0.24833
ALA	75	to ALA energy chang	0
VAL	76	to ALA energy chang	-0.32493
ALA	77	to ALA energy chang	0
GLN	78	to ALA energy chang	-0.70288
LYS	79	to ALA energy chang	-0.82435
THR	80	to ALA energy chang	0.01386
VAL	81	to ALA energy chang	-0.22118
GLU	82	to ALA energy chang	-0.64199
GLY	83	to ALA energy chang	-0.8238
ALA	84	to ALA energy chang	0
GLY	85	to ALA energy chang	-0.68118
SER	86	to ALA energy chang	-0.37455

ILE	87	to ALA energy chang	-0.06312
ALA	88	to ALA energy chang	0
ALA	89	to ALA energy chang	0
ALA	90	to ALA energy chang	0
THR	91	to ALA energy chang	-0.72303
GLY	92	to ALA energy chang	1.04951
PHE	93	to ALA energy chang	0.172081
VAL	94	to ALA energy chang	-0.21222
LYS	95	to ALA energy chang	0.058828
LYS	96	to ALA energy chang	0.391063
ASP	97	to ALA energy chang	0.259945
GLN	98	to ALA energy chang	-1.58197

The Van der Waal's Results of H50Q

ALA	40 to ALA ε	0
GLY	41 to ALA ε	-0.08436
GLY	42 to ALA ε	-0.29343
THR	43 to ALA ε	-0.02007
ALA	44 to ALA ε	0
ALA	45 to ALA ε	0
GLY	46 to ALA ε	-0.45168
VAL	47 to ALA ε	-0.3363
VAL	48 to ALA ε	-0.25847
H1S	49 to ALA ε	-1.01113
GLY	50 to ALA ε	-0.82955
VAL	51 to ALA ε	-0.09819
THR	52 to ALA ε	-1.33956
THR	53 to ALA ε	-1.03373
VAL	54 to ALA ε	-0.50058
ALA	55 to ALA ε	0
GLU	56 to ALA ε	-2.1632
LYS	57 to ALA ε	-1.73159
THR	58 to ALA ε	-0.77455
LYS	59 to ALA ε	-2.04662
GLU	60 to ALA ε	-2.45588
GLN	61 to ALA ε	-1.14301
VAL	62 to ALA ε	-0.10289
THR	63 to ALA ε	-0.81446
ASN	64 to ALA ε	-0.40699
VAL	65 to ALA ε	-0.30524
GLY	66 to ALA ε	0.149623
GLY	67 to ALA ε	-0.53156
ALA	68 to ALA ε	0
VAL	69 to ALA ε	-0.20203
VAL	70 to ALA ε	-0.15095
THR	71 to ALA ε	-0.07119
GLY	72 to ALA ε	-1.04658
VAL	73 to ALA ε	0.114624
THR	74 to ALA ε	-0.13655
ALA	75 to ALA ε	0
VAL	76 to ALA ε	-0.46914
ALA	77 to ALA ε	0
GLN	78 to ALA ε	-0.52335
LYS	79 to ALA ε	-0.90445
THR	80 to ALA ε	-0.0704
VAL	81 to ALA ε	-0.44409
GLU	82 to ALA ε	-0.90469
GLY	83 to ALA ε	-0.76488
ALA	84 to ALA ε	0
GLY	85 to ALA ε	-0.67519
SER	86 to ALA ε	-0.50655

ILE	87 to ALA є	0.126322
ALA	88 to ALA є	0
ALA	89 to ALA є	0
ALA	90 to ALA є	0
THR	91 to ALA є	0.227571
GLY	92 to ALA є	0.747755
PHE	93 to ALA є	0.519248
VAL	94 to ALA є	-0.60275
LYS	95 to ALA є	-0.95343
LYS	96 to ALA є	0.405742
ASP	97 to ALA є	-0.51859
GLN	98 to ALA є	-1.15573

The Van der Waal's results of A53T

VAL	40	to ALA ene	-0.31344
GLY	41	to ALA ene	-0.17645
SER	42	to ALA ene	-0.67931
LYS	43	to ALA ene	-2.23915
THR	44	to ALA ene	-1.64853
LYS	45	to ALA ene	-1.16613
GLU	46	to ALA ene	-0.84287
GLY	47	to ALA ene	-0.71218
VAL	48	to ALA ene	-0.89191
VAL	49	to ALA ene	-0.36514
H2S	50	to ALA ene	-1.01243
GLY	51	to ALA ene	-0.6424
VAL	52	to ALA ene	-0.4803
ALA	53	to ALA ene	0
THR	54	to ALA ene	-1.6859
VAL	55	to ALA ene	-0.33464
ALA	56	to ALA ene	0
GLU	57	to ALA ene	-1.77895
LYS	58	to ALA ene	-1.00086
THR	59	to ALA ene	-1.09602
LYS	60	to ALA ene	-1.29367
GLU	61	to ALA ene	-1.12575
GLN	62	to ALA ene	-0.32765
VAL	63	to ALA ene	-0.38133
THR	64	to ALA ene	-0.41597
ASN	65	to ALA ene	-0.19075
VAL	66	to ALA ene	-0.39737
GLY	67	to ALA ene	0.24876
GLY	68	to ALA ene	-0.56118
ALA	69	to ALA ene	0
VAL	70	to ALA ene	0.077646
VAL	71	to ALA ene	-0.11031
THR	72	to ALA ene	-0.65855
GLY	73	to ALA ene	-0.67939
VAL	74	to ALA ene	-0.34955
THR	75	to ALA ene	-0.77111
ALA	76	to ALA ene	0
VAL	77	to ALA ene	-0.40986
ALA	78	to ALA ene	0
GLN	79	to ALA ene	-0.49127
LYS	80	to ALA ene	-1.3096
THR	81	to ALA ene	0.105394
VAL	82	to ALA ene	-0.05561
GLU	83	to ALA ene	-0.5639
GLY	84	to ALA ene	-0.84575
ALA	85	to ALA ene	0
GLY	86	to ALA ene	-0.69522

SER	87	to ALA ene	-0.53425
ILE	88	to ALA ene	-0.11223
ALA	89	to ALA ene	0
ALA	90	to ALA ene	0
ALA	91	to ALA ene	0
THR	92	to ALA ene	-0.35856
GLY	93	to ALA ene	0.256498
PHE	94	to ALA ene	0.327564
VAL	95	to ALA ene	-0.43316
LYS	96	to ALA ene	-0.31992
LYS	97	to ALA ene	-0.08712
ASP	98	to ALA ene	-0.17872

The Van der Waal's results of A30P

T2. The Van der Waal's results of H50Q, A53T and A30P are listed.
