# Supplementary material for: Studying the effect of alpha-synuclein and Parkinson’s disease linked mutants on inter pathway connectivities
Source: Sci Rep. 2021 Aug 11;11:16365. doi: 10.1038/s41598-021-95889-5 (PMC8358055; doi:10.1038/s41598-021-95889-5)
Supplement: Supplementary file 5 — Supplementary Information 5. [file 41598_2021_95889_MOESM5_ESM.pdf]

Parkinson disease	0.090884
Alzheimer disease	0.093325
Cocaine addiction	0.109259
Mitophagy	0.085275
Amphetamine addiction	0.118707
Synaptic vesicle cycle	0.109259
Serotonergic synapse	0.092579
Dopaminergic synapse	0.109259
Alcoholism	0.109259
MAPK signaling pathway	0.082192

WT-Huma

Parkinson’s Disease	0.168757
Parkin-Ubiquitin Proteasomal System pathway	0.142153
Dopaminergic	0.131422
Monoamine Transport	0.131422
Synaptic Vesicle Pathway	0.143444
Alzheimer's Disease	0.140411
Androgen receptor signaling pathway	0.142391

G51D-slow

Parkinson’s Disease	0.151512
Cocaine addiction	0.162626
Mitophagy -animal	0.132733
Amphetamine addiction	0.093464
Dopaminergic	0.162626
Alcoholism	0.162626
Alzheimer's Disease	0.134414

E46K-fast

Parkinson’s Disease	0.134929
Alzheimer disease	0.112156
Cocaine addiction	0.159189
Mitophagy -animal	0.116161
Amphetamine addiction	0.159189
Dopaminergic	0.159189
Alcoholism	0.159189

H50Q

Parkinson disease	0.092693
Alzheimer disease	0.095265
MAPK signaling pathway	0.086225
Cocaine addiction	0.110642
Mitophagy	0.08683
Amphetamine addiction	0.110642
Synaptic vesicle cycle	0.110642
Serotonergic synapse	0.085778
Dopaminergic synapse	0.110642
Alcoholism	0.110642

A53T

Parkinson disease	0.067995
Cocaine addiction	0.101673
Amphetamine addiction	0.101673
Dopaminergic synapse	0.101673
Alzheimer disease	0.068176
Alcoholism	0.101673
Folate biosynthesis	0.094873
Tyrosine metabolism	0.094873
Prolactin signaling pathway	0.095251
Synaptic vesicle cycle	0.043813
Serotonergic synapse	0.06583
MAPK signaling pathway	0.062499

A30P

T3. A table of each mutation of alpha-synuclein along with their PR score
